# The ovarian cancer oncobiome

**DOI:** 10.18632/oncotarget.16717

**Published:** 2017-03-30

**Authors:** Sagarika Banerjee, Tian Tian, Zhi Wei, Natalie Shih, Michael D. Feldman, James C. Alwine, George Coukos, Erle S. Robertson

**Affiliations:** ^1^ Department of Otorhinolaryngology-Head and Neck Surgery, University of Pennsylvania, Philadelphia, Pennsylvania, United States of America; ^2^ Department of Computer Science, New Jersey Institute of Technology, Newark, New Jersey, United States of America; ^3^ Department of Pathology and Laboratory Medicine, University of Pennsylvania, Philadelphia, Pennsylvania, United States of America; ^4^ Department of Cancer Biology, University of Pennsylvania, Philadelphia, Pennsylvania, United States of America; ^5^ Department of Oncology, University Hospital of Lausanne (CHUV), Lausanne, Switzerland

**Keywords:** oncobiome, microbiome, ovarian cancer, pathochip, next generation sequencing

## Abstract

Humans and other mammals are colonized by microbial agents across the kingdom which can represent a unique microbiome pattern. Dysbiosis of the microbiome has been associated with pathology including cancer. We have identified a microbiome signature unique to ovarian cancers, one of the most lethal malignancies of the female reproductive system, primarily because of its asymptomatic nature during the early stages in development. We screened ovarian cancer samples along with matched, and non-matched control samples using our pan-pathogen array (PathoChip), combined with capture-next generation sequencing. The results show a distinct group of viral, bacterial, fungal and parasitic signatures of high significance in ovarian cases. Further analysis shows specific viral integration sites within the host genome of tumor samples, which may contribute to the carcinogenic process. The ovarian cancer microbiome signature provides insights for the development of targeted therapeutics against ovarian cancers.

## INTRODUCTION

In the US, ovarian cancer is the second most common and most deadly of the gynecologic cancers, affecting 1 in 70 women, with a mortality rate of 1% of all women (http://www.merckmanuals.com/professional/gynecology-and-obstetrics/gynecologic-tumors/ovarian-cancer). This accounts for its being the 5th leading cause of cancer-related deaths in women, causing an estimated 22,280 new cases (1.3% of all new cancer cases) and 14,240 deaths (2.4% of all cancer deaths) in 2016 (www.cancer.org). Importantly, the incidence is even higher in developed countries (http://www.wcrf.org). Due to the asymptomatic nature of the early stage of the disease most patients go undiagnosed until the cancer reaches an advanced stage [1]. Thus finding specific biomarkers for early diagnosis of the disease is of utmost importance. Many studies have found that DNA of the Human Papillomavirus (HPV)-16 and HPV-18 is associated with ovarian carcinomas [2–5]. However, recent studies from our laboratory and others [6–8] have found that the tumor microbiome may be far more complex. We have defined unique microbial signatures associated with triple negative breast cancer and head and neck cancer [6] (Banerjee *et al*., unpublished). These signatures potentially provide insight into predisposition, presence or prognosis of the cancer. Such diagnostic data may increase the therapeutic potential for early detection and treatment.

In the present study we used the PathoChip, a microarray-based approach comprised of probes for detection of all known viruses and other human pathogenic microorganisms [6, 9]. The current version of the PathoChip contains 60,000 probes representing all known viruses, 250 helminths, 130 protozoa, 360 fungi and 320 bacteria [6, 9]. In addition to probes that identify specific viruses and micro-organisms, PathoChip also contains family-specific conserved probes which provide a means for detecting previously uncharacterized members of a family. Using this technique we have previously identified a microbiome signature associated with triple negative breast cancers [6], and oropharyngeal squamous cell carcinomas (Banerjee *et al*., unpublished).

We have used 99 ovarian cancer samples and 20 matched (tissue adjacent to the tumor deemed non-cancerous by pathological analysis) and 20 unmatched control samples to define a specific ovarian cancer microbiome signature which is distinct from the signature of the controls. To corroborate these results we selected microbial probes across the different organisms detected by the PathoChip screen and used them to capture the signatures from the ovarian cancer samples. This enrichment allowed targeted next generation sequencing to validate the PathoChip screen results and also allowed us to identify microbial insertion sites in the host genome of the ovarian cancer tissues. The data generated in this study suggest a robust and specific microbiome associated with ovarian cancer. Whether or not these organisms contribute as direct drivers to the cancer or simply persist as bystanders or secondary in a supportive tumor microenvironment remains to be determined.

## RESULTS

### Microbial signatures uniquely associated with ovarian cancer

We used the PathoChip technology to screen ovarian cancer samples, as well as matched and non-matched controls. To establish the microbiome signatures we compared the average hybridization signal for each probe in the cancer samples versus the controls. Those probes that detected significant hybridization signals in the cancer samples (*p*-value < 0.05, log fold change in hybridization signal > log1), were considered. Additionally, we calculated the percent prevalence of the specific microbial signatures in the cancer samples, these data indicate how prevalent a significant virus or microorganism signature is in the cancer samples regardless of the hybridization intensity. Similarly, we also detected microbiome signatures in the matched and non-matched control samples versus the ovarian cancer samples. The signature of non-matched controls is quite distinct while there is more similarity between the tumor tissue and the matched controls. However, there are distinct viral and microbial signatures in the tumor-specific signature.

### Viral signatures associated with ovarian cancer

**Figure 1:**
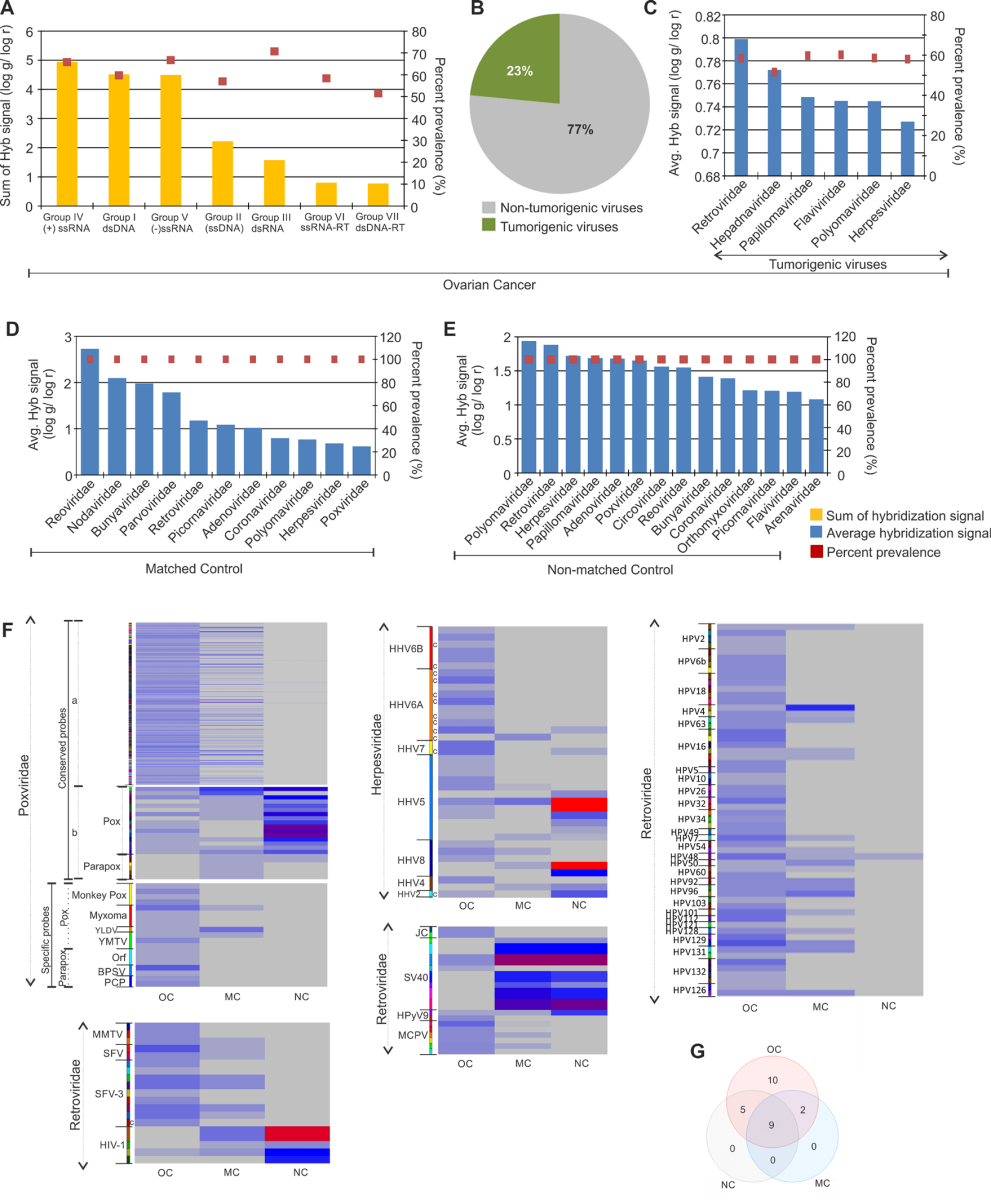
Viral signatures detected in ovarian, matched and non-matched controls (**A**) Molecular signatures of viral groups detected in ovarian cancer, with the total hybridization signal for each viral groups (sum of average hybridization signal for all the representative families in the group) represented according to descending order as a bar graph and prevalence of the same as dots. (**B**) The percentage of tumorigenic viral signatures detected in the ovarian cancers are represented in a pie chart. (**C**) The average hybridization signal of the tumorigenic viral signatures detected in the ovarian cancers are represented in the decreasing order as a bar graph, whereas their respective prevalence are represented as dots. (**D** and **E**) The signatures of viral families detected in matched (D) and non-matched (E) controls are represented according to decreasing average hybridization signals as bar graphs, and their respective prevalence as dots. (**F**) Heat map of average hybridization signals for probes of Poxviruses, Retroviruses, Herpesviruses, Polyomaviruses and Papillomaviruses detected in ovarian cancers (OC), matched (MC) and non-matched (NC) controls. Heat map of average hybridization signal of both conserved and specific probes of Poxviridae are shown. Among the conserved poxviridae probes mentioned, (a) comprises the conserved probes detected significantly in the ovarian cancer versus the controls, and (b) comprises the conserved probes detected significantly in the controls versus the ovarian cancers screened. In the heat map with Herpesviridae probes, those mentioned (c) are conserved probes. All other probes in these heat maps are specific probes. (**G**) Venn diagram showing the number of viral families common or unique to the ovarian cancer and control samples.

The viral signatures detected in the ovarian cancer and control samples are shown according to their decreasing hybridization signal along with their prevalence in Figure 1A–1E. By summing all of the hybridization signals for viral families we found that the predominant signatures detected in the ovarian cancers were positive sense single stranded RNA viruses, double stranded DNA viruses and negative sense single stranded RNA viruses (Figure 1A). Among the signatures for viral families detected, 23% were identified as tumorigenic viruses (Figure 1B), and were prevalent on average, in more than 50% of the cancer samples screened (Figure 1C). Signatures of Retroviridae showed the highest hybridization signal, followed by that of Hepadnaviridae, Papillomaviridae, Flaviviridae, Polyomaviridae and Herpesviridae (Figure 1C). Notably, Papillomaviridae family members have previously been shown to be associated with ovarian cancer [2, 10]. Interestingly, we found papilloma virus signatures in the cancer samples and in the non-matched controls, but not at significant levels in the matched controls. The papilloma virus signatures in the ovarian cancer samples screened included not only HPV16 and 18 but also other HPVs (HPV-2, 4, 5, 6b, 7, 10, 32, 48, 49, 50, 60, 54, 92, 96, 101, 128, 129, 131, 132) (Figure 1F). However the HPV signatures in matched controls that showed significantly high hybridization signal intensity over those in cancer samples, were HPV 41, 88, 53 and 103 (Figure 1F). We also found an abundance of other viral signatures in the ovarian cancer samples (Table 1, Figure 1F, and Supplementary Figure 1), including Herpesviridae (HHV4, HHV8, HHV5, HHV6a, HHV 6b), Poxviridae (both pox and parapoxvirus), Polyomaviridae (Merkel cell polyomavirus, JC polyomavirus, Simian virus 40), Retroviridae (Simian foamy virus, Mouse mammary tumor virus).

**Table 1:** Microbial signatures detected in ovarian cancer and control samples

	Cancer	MC	NC	Cancer/MC	Cancer/NC	Cancer/MC/NC
Viral signatures	Anelloviridae Astroviridae Birnaviridae Bornaviridae Caliciviridae Hepadnaviridae Iridoviridae Paramyxoviridae Rhabdoviridae Togaviridae			Nodaviridae Parvoviridae	Arenaviridae Circoviridae Flaviviridae Orthomyxoviridae Papillomaviridae	Adenoviridae Bunyaviridae Coronaviridae Herpesviridae Picornaviridae Polyomaviridae Poxviridae Reoviridae Retroviridae
Bacterial signatures	**Proteobacteria**: *Aeromonas* *Agrobacterium* *Anaplasma* *Arcobacter* *Bartonella* *Brucella* *Burkholderia* *Campylobacter* *Coxiella* *Francisella* *Helicobacter* *Klebsiella* *Legionella* *Methylobacterium* *Neisseria* *Orientia* *Pasteurella* *Proteus* *Pseudomonas* *Rickettsia* *Shewanella* *Shigella* *Sphingomonas* *Stenotrophomonas* *Vibrio* *Wolbachia* *Yersinia* **Firmicutes**: *Abiotrophia* *Bacillus* *Enterococcus* *Erysipelothrix* *Geobacillus* *Lactobacillus* *Lactococcus* *Listeria* *Pediococcus* *Peptoniphilus* *Staphylococcus* **Bacteroidetes**: *Bacteroides* *Flavobacterium* *Porphyromonas* *Prevotella* *Actinobacteria*: *Corynebacterium* *Propionibacterium* **Chlamydiae**: *Chlamydia* *Chlamydophila* *Fusobacteria*: *Fusobacterium* *Streptobacillus* **Spirochaetes**: *Leptospira* *Treponema* **Tenericutes**: *Mycoplasma* *Ureaplasma*	**Proteobacteria**: *Morganella*	**Proteobacteria**: *Brevundimonas* *Campylobacter*	**Proteobacteria**: *Azorhizobium* *Escherichia* *Firmicutes*: *Clostridium*	**Proteobacteria**: *Bordetella* *Salmonella* *Bacteroidetes*: *Chryseobacterium* *Actinobacteria*: *Mycobacterium* *Firmicutes*: *Streptococcus*	
Fungal signatures	*Acremonium* *Ajellomyces* *Aspergillus* *Candida* *Cladosporium* *Coccidioides* *Cryptococcus* *Cunninghamella* *Issatchenkia* *Nosema* *Paracoccidioides* *Penicillium* *Pleistophora* *Pneumocystis* *Rhizomucor* *Rhizopus* *Rhodotorula* *Trichophyton*	*Exophiala* *Phialophora*		*Alternaria* *Malassezia* *Mucor* *Trichosporon*	*Absidia* *Cladophialophora* *Fusarium*	*Geotrichum*
Parasitic signatures	*Ancylostoma* *Anisakis* *Armillifer* *Ascaris* *Babesia* *Balantidium* *Bipolaris* *Blastocystis* *Capillaria* *Dicrocoelium* *Dipylidium* *Echinococcus* *Echinostoma* *Entamoeba* *Enterobius* *Hartmannella* *Heteroconium* *Hymenolepis* *Leishmania* *Loa* *Metagonimus* *Necator* *Onchocerca* *Plasmodium* *Sarcocystis* *Schistosoma* *Strongyloides* *Toxascaris* *Toxocara* *Trichomonas* *Trichuris* *Wuchereria*	*Prosthodendrium*		*Acanthamoeba* *Naegleria* *Taenia* *Trichinella*	*Contracaecum* *Diphyllobothrium*	

MC: Matched Control, NC: Non-matched Control.

In the adjacent matched controls and in non-matched control samples, we also detected signatures of tumorigenic viral families, along with other viral signatures (Figure 1D and 1E). Figure 1G and Table 1 shows the common as well as unique viral signatures detected in ovarian cancer, when compared to the matched and non-matched controls.

The data suggest a substantial perturbation of the virome in ovarian cancer. First, the average hybridization signal for the viral families detected in the cancer is actually lower compared to the control samples (compare Supplementary Figure 1 with Figure 1C–1E); Second, despite lower hybridization signal for many viruses in the cancer samples, the viral families present are quite different from controls; for example, signatures of Anelloviridae, Astroviridae, Birnaviridae, Bornaviridae, Caliciviridae, Hepadnaviridae, Iridoviridae, Paramyxoviridae, Rhabdoviridae and Togaviridae were detected at significant levels only in the cancer samples (Supplementary Figure 1, Table 1). Third, among the viral families detected in both cancer and control samples, specific members of a virus family differed between cancer and controls. For example, specific molecular signatures of the high risk HPV16 and 18 were detected only in the cancer samples and not in the matched or non-matched control group. Instead the non-matched control samples showed significant detection of molecular signatures of the L1 major capsid gene of HPV 41, 88, 53, and E1 gene of HPV 103 (Figure 1F and Supplementary Table 2). A similar situation was detected with the poxviridae. While signatures of poxviridae that are conserved across the family were significantly detected in cancer as well as the controls (both matched and non-matched) (Figure 1F, Supplementary Table 2), highly specific signatures of certain poxviruses [Monkeypox virus, Myxoma virus, Yaba monkey tumor virus (YMTV), Yaba-like disease virus (YLDV)] and parapoxviruses [(Pseudocowpox virus (PCP), Orf virus (Orf), Bovine papular stomatitis virus (BPSV)] were detected only in the ovarian cancer samples (Figure 1F, Supplementary Table 2). The specific parapoxvirus signatures detected were that of IL-10 encoded by Orf virus and Bovine papular stomatitis virus, and the A-type inclusion protein of Pseudocowpox virus and Orf virus, as well as the glycoprotein of Orf virus (Supplementary Table 2). Specific signatures of poxviruses detected were sequences of thymidine kinase (66R) and ankyrin repeat (147R) of the tumorigenic Yaba monkey tumor virus, 3-beta-hydroxysteroid dehydrogenase of Yaba-like disease virus (Supplementary Table 2). Also, the majority of the Polyomavirus probes significantly detected in the ovarian cancers were that of Merkel cell Polyomaviruses which were undetectable in the controls, whereas the majority of the Polyomavirus probes detected in the controls were that of SV40, traces of which were also detected in the cancers (Figure 1F, Supplementary Table 2). Among the retroviral probes detected in the majority of cancers were specific probes of Mammary Tumor Virus (MMTV) and Foamy Virus (SFV), whereas, the majority of Retroviral probes detected in the controls were specific probes for the lentivirus subgroup of retroviruses (Figure 1E, Supplementary Table 2). Interestingly, the detection of Herpesviridae probes identified HHV2 with high significance in the non-matched control compared to the cancers. However, the cancer samples showed detection for conserved and specific probes of HHV6A and HHV6B which were undetectable in the controls. Other herpesviridae probes of HHV4, HHV5 and HHV8 were detected in both cancer and non-matched control samples (Figure 1F, Supplementary Table 2).

The data as a whole suggest that specific viral signatures are dramatically altered in the cancer tissue. Some signatures appear only in the cancer or have significantly increased hybridization intensity, while others are decreased compared to the surrounding tissue. Several points must be kept in mind when considering these data: 1) the tumor microenvironment may provide advantages for the persistence of some viruses, thus promoting their presence in the cancer. Hence, their presence need not be related to the cause of the cancer. Similarly, the appearance of a virus in the matched control and not the cancer may suggest that the tumor microenvironment is inhibitory for persistence of the virus. 2) The probes may also be detecting relatives or variants of known viruses from which the probes were derived. For example, specific probes for lentiviruses including HIV-1 were positive in the analysis of control samples. These are de-identified samples; however we doubt that these patients were HIV positive but suspect that the probes are likely detecting the presence of a related, uncharacterized human lentivirus.

### Identification of bacterial signatures associated with ovarian cancer

**Figure 2:**
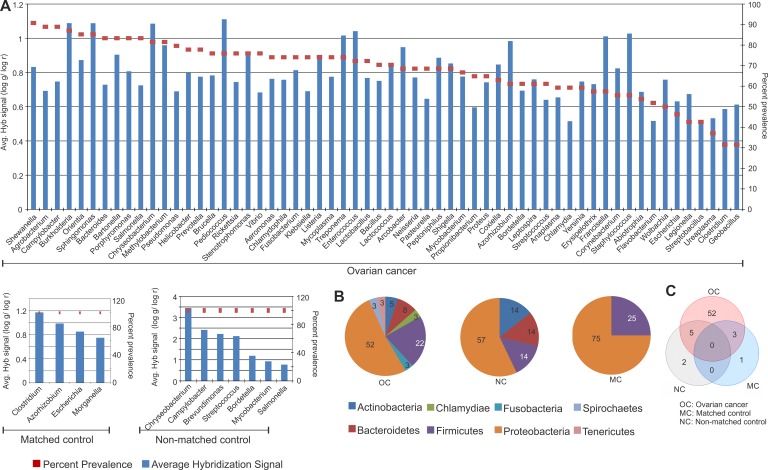
Bacterial signatures detected in ovarian, matched and non-matched controls (**A**) Bacterial signatures detected in ovarian cancers, matched and non-matched controls. The prevalence of those signatures are represented in the decreasing order as dots, and their average hybridization signal being represented as a bar graph. (**B**) Distribution of bacterial phyla detected in ovarian cancer, matched and non-matched controls. (**C**) Venn diagram showing the number of bacteria common or unique to the ovarian cancer and control samples.

Similar to that seen with the viruses, the bacterial signatures of the tumor tissue were dramatically altered from those of matched and non-matched controls. The specific bacterial signatures detected in the cancer and the matched and non-matched samples are shown in Figure 2A according to their decreasing prevalence. Two predominant bacterial phyla were detected in the ovarian cancer samples screened. They were Proteobacteria (52%), followed by Firmicutes (22%) (Figure 2B). We also detected other phyla at lower percentages including Bacteroidetes, Actinobacteria, Chlamydiae, Fusobacteria, Spirochaetes and Tenericutes in the cancer samples. Signatures of Proteobacteria and Firmicutes were also detected significantly in the matched control samples screened, and that of Proteobacteria, Actinobacteria, Bacteroidetes and Firmicutes were detected significantly in the non-matched control samples (Figure 2B). Many more bacterial signatures were significantly detected in the cancer samples compared to the controls. The signatures associated only with the ovarian cancer samples are listed Table 1). The different bacterial signatures, unique or common to the control and ovarian cancer samples are listed in Table 1 and represented in Figure 2C.

While signatures of Pediococcus was detected with the highest hybridization signal in the ovarian cancer samples screened, followed closely by that of Burkholderia, Sphingomonas, Chryseobacterium, Enterococcus, Staphylococcus, Treponema and Francisella [(log g/log r) > 1], Shewanella signatures were detected with the highest prevalence in 91% of the cancers (Figure 2A). The majority of the bacterial signatures detected in the cancers had high prevalence, except for signatures of Escherichia, Legionella, Streptobacillus, Ureaplasma, Clostridium, Geobacillus which were detected in less than 50 percent of the cancer samples screened (Figure 2A). Interestingly, there are no common bacteria between all 3 types of samples (Figure 2C, Table 1). However, 5 agents were shared between the cancer and non-matched controls, and 3 agents between the cancer and matched controls (Figure 2C, Table 1). 52 unique bacterial agents were detected predominantly in only the cancer (Figure 2C, Table 1).

### Identification of fungal signatures associated with ovarian cancer

**Figure 3:**
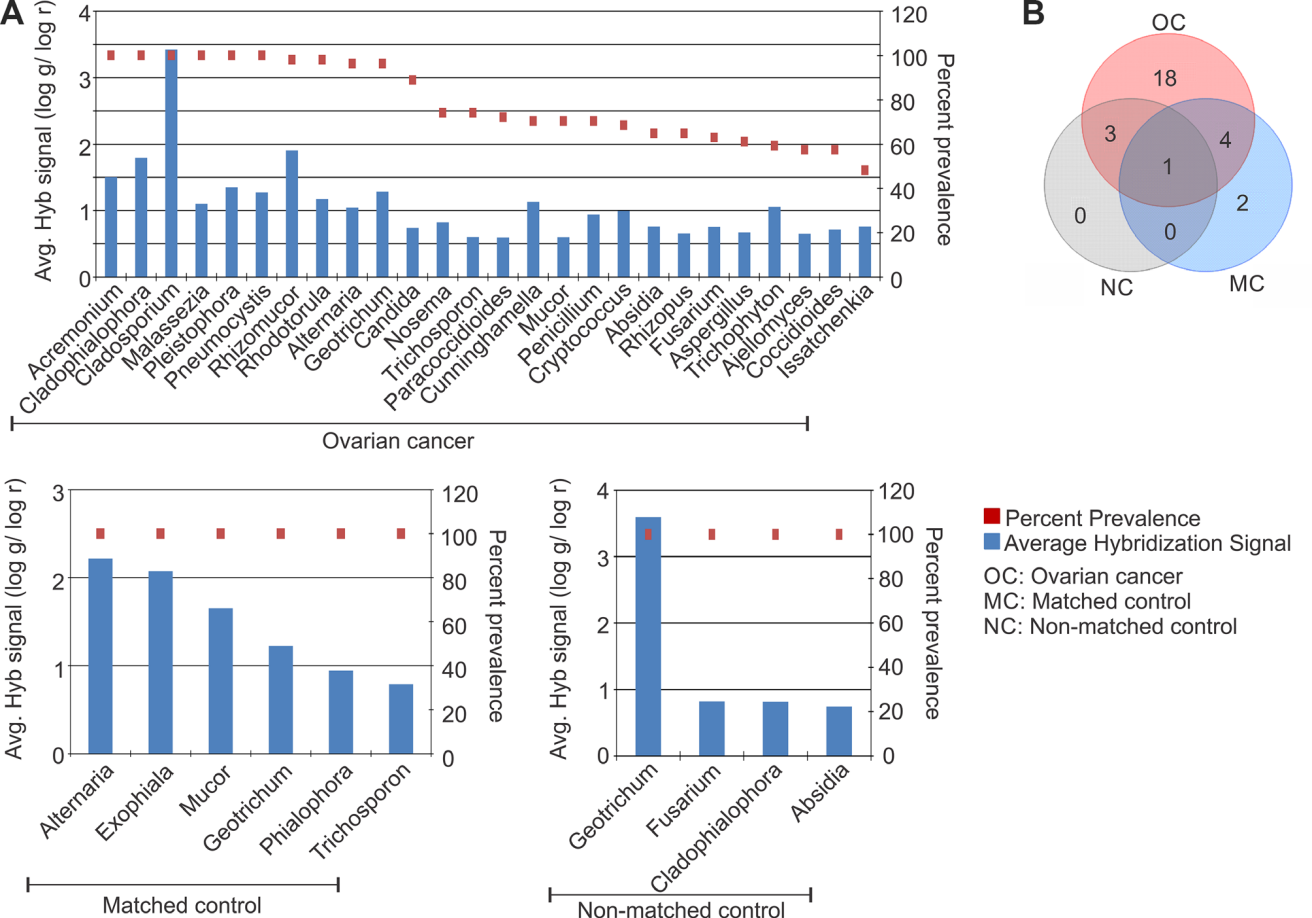
Fungal signatures detected in ovarian, matched and non-matched controls (**A**) Fungal signatures detected in ovarian cancer, matched and non-matched controls. The prevalence of those signatures are represented in the decreasing order as dots, and their average hybridization signal being represented as a bar graph. (**B**) Venn diagram showing the number of fungi common or unique to the ovarian cancer and control samples.

Our pathogen screen for fungal signatures again suggests a significant perturbation of the microbiome in the tumor. The fungal signatures detected in the ovarian cancer and controls are shown according to their decreasing prevalence in Figure 3A. The 18 fungal signatures that were detected only in the ovarian cancer samples and interestingly not found associated with the controls are listed (Table 1, Figure 3B). 18S rRNA signatures of *Cladosporium* were detected in all the ovarian cancer samples with the highest hybridization signal (Figure 3A). Signatures of *Pneumocystis*, *Acremonium, Cladophialophora*, *Malassezia* and Microsporidia *Pleistophora* were also detected significantly in all the ovarian cancer samples screened (Figure 3A). Signatures of *Rhizomucor*, *Rhodotorula*, *Alternaria*, *Geotrichum* were found to be associated with more than 95% of the ovarian cancer samples screened (Figure 3A). It should be noted that the signature of *Geotrichum* was also detected in all the control samples (Table 1 and Figure 3A). Therefore the associated fungal agents appear to be dominant in the ovarian cancer with only *Geotrichum* common among the cancer and controls. This suggests that the fungal signatures may be more tightly associated in this particular microenvironment than previously predicted.

### Identification of parasitic signatures associated with ovarian cancer

**Figure 4:**
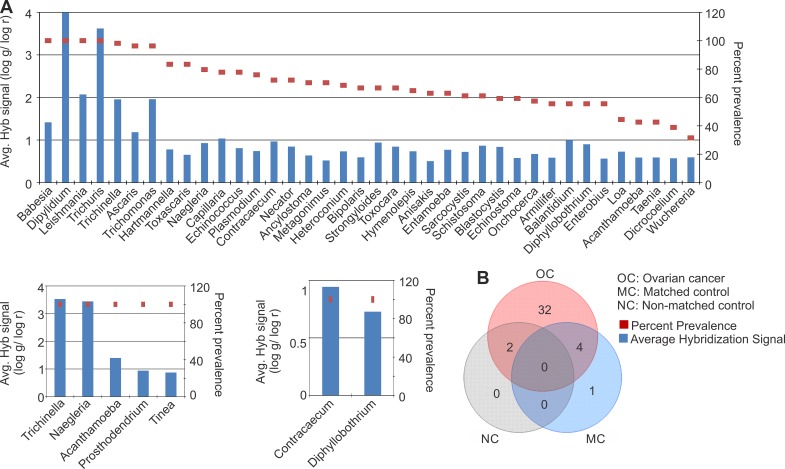
Parasitic signatures detected in ovarian, matched and non-matched controls (**A**) Parasitic signatures detected in ovarian cancer, matched and non-matched controls. The prevalence of those signatures are represented in the decreasing order as dots, and their average hybridization signal being represented as a bar graph. (**B**) Venn diagram showing the number of parasites common or unique to the ovarian cancer and control samples.

The parasitic signatures detected in the ovarian cancer and controls are shown (Figure 4A), according to their decreasing prevalence. The parasitic signature significantly detected in cancer samples was far more complex than the matched and, especially, the non-matched controls, once again suggesting a marked perturbation of the tumor microbiome. The parasitic signatures detected only in the ovarian cancer samples are listed (Figure 4B, Table 1). All of the tumor samples showed a high hybridization signal (log g/log r > 2) for the 28S rRNA signature of *Dipylidium*. A high hybridization signal for the 18S rRNA signatures of *Trichuris* and *Leishmania* was also found in all of the ovarian cancer samples (Figure 4A). The 18S rRNA signatures of *Babesia* were also significantly detected in all the ovarian cancer samples, although with a relatively moderate hybridization signal (log g/log r > 1, < 2) (Figure 4A). 18S rRNA signatures of *Trichinella*, *Ascaris*, and *Trichomonas* were detected in >95% of the ovarian cancer samples screened, also with a moderate hybridization signal intensity (log g/log r > 1, < 2) (Figure 4A). The other parasitic signatures detected in the ovarian cancer listed in Figure 4A were detected with lower hybridization signal intensity (log g/log r < 1), although with high prevalence except for signatures of *Loa loa*, *Acanthamoeba*, *Taenia*, *Dicrocoelium*, Wuchereria which were detected in less than 45% of the ovarian cancer samples screened. Signatures of 4 parasites that were detected in the cancer samples were also found in the adjacent matched control samples; these include *Acanthamoeba*, *Naegleria*, *Taenia* and *Trichinella* (Figure 4A, Table 1). However, they were not detected in the non-matched controls (Figure 4A).

### Hierarchical clustering of the ovarian cancer samples

**Figure 5:**
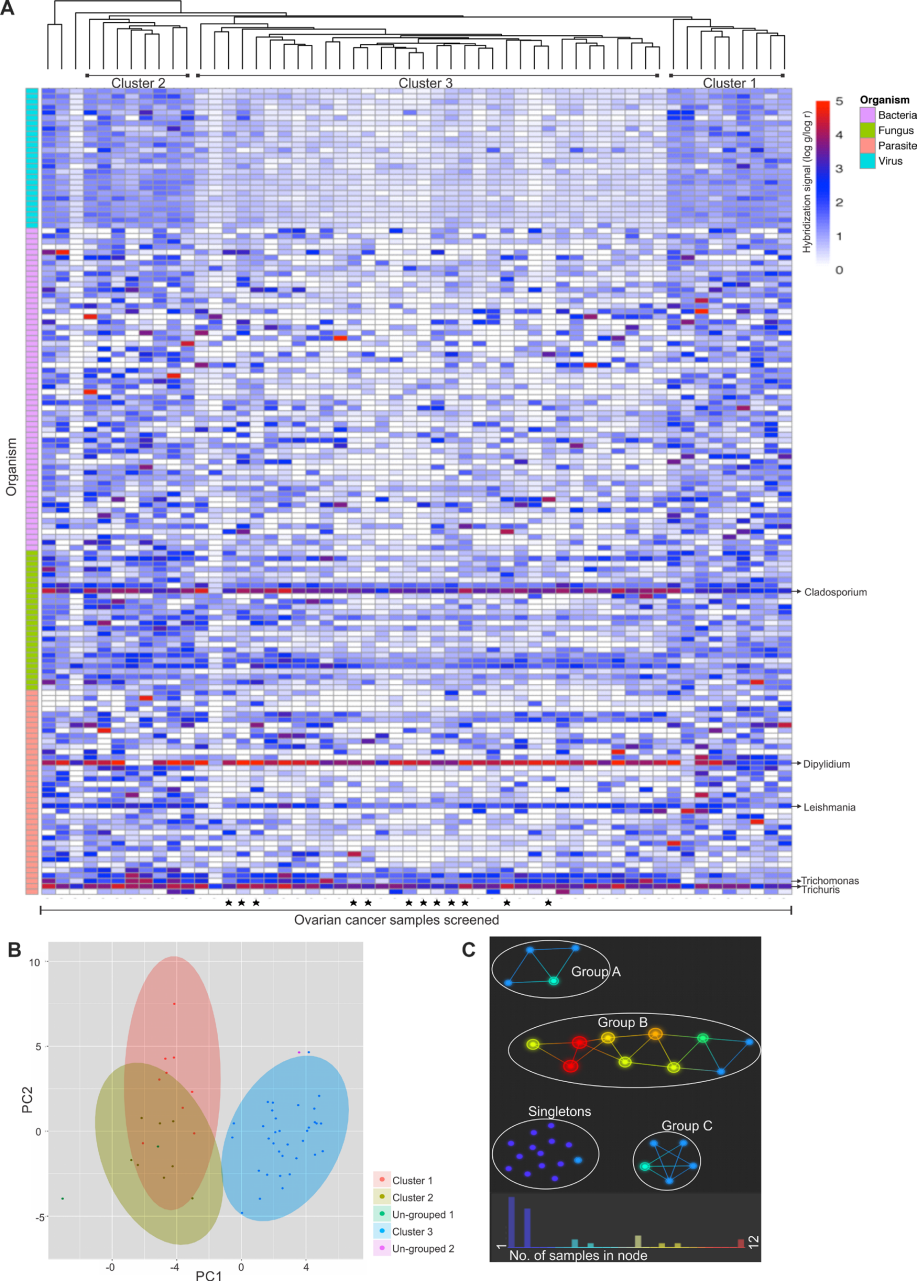
Hierarchical clustering of ovarian cancer samples screened Hierarachial clustering of 99 ovarian cancer samples. (**A**). Hierarchial clustering by R program using Euclidean distance, complete linkage and non-adjusted values. Samples marked (▪) were the samples that were screened in pools, rest were screened individually. (**B**). Clustering of the OSCC samples using NBClust software [CH (Calinski and Harabasz) index, Euclidean distance, complete linkage]. (**C**). Topological analysis using Ayasdi software, using Euclidean (L2) metric and L-infinity centrality lenses. The cancer samples that had similar detection for viral and microbial signatures formed the nodes, and those nodes are connected by an edge if the corresponding node have detection pattern in common to the first node. Each nodes are colored according to the number of samples clustered in each node.

Hierarchical clustering analysis compares the similarity of the overall microbiome signatures detected in each ovarian cancer sample and clusters the samples together based on common microbiome similarity (Figure 5A–5B). While some samples did not group into a cluster (namely un-grouped 1 and 2) (Figure 5B), majority of the samples grouped into three distinct clusters, namely cluster 1, 2 and 3 (Figure 5A and 5B), with cluster 3 samples showing significant differences in detection of several viral and other microbial signatures compared to the samples of cluster 1 and 2. Supplementary Table 3 shows the significant differences in microbial detection between the clusters. Ovarian cancer samples of cluster 1 and 2 showed significant differences in the detection of 2 viral agents (Arenaviridae and Flaviviridae) and bacterial agents (*Coxiella* and *Listeria*) signatures, and few fungal (*Acremonium*, *Cladosporium*, *Mucor*, *Pleistophora*, *Pneumocystis* and *Rhodotorula*) and parasitic (*Babesia*, *Dipylidium*, *Leishmania*, *Toxocara*, *Trichinella*, *Trichomonas* and *Trichuris*) signatures. These signatures are all of higher intensities in cluster 2 than 1. On the other hand, ovarian cancer samples of cluster 3 had significantly less detection of almost all the viral and several microbial signatures mentioned in Supplementary Table 3.

Based on topological analysis, the ovarian cancer samples clustered into 3 groups (A, B and C), while some could not be grouped together (singletons) (Figure 5C). Supplementary Table 4 shows significant differences in microbial detection in each groups. Group B had significantly higher detection of the following signatures compared to Group A: viral signatures of Coronaviridae, Astroviridae, Togaviridae, Reoviridae, Papillomaviridae, Poxviridae, Bunyaviridae, Picornaviridae, Paramyxoviridae, Bornaviridae, Birnaviridae, Rhabdoviridae, Caliciviridae, Arenaviridae and Flaviviridae; along with certain bacterial signatures of *Porphyromonas*, *Anaplasma*, *Azorhizobium*, *Corynebacterium*, *Arcobacter*, *Lactococcus*, *Methylobacterium*, *Shigella*, *Proteus*, *Brucella*, *Ureaplasma* and *Prevotella*; fungal signatures of *Absidia*, *Trichophyton*, *Ajellomyces*, *Geotrichum* and *Candida*;and parasitic signatures of *Ascaris*, *Bipolaris*, *Acanthamoeba*, *Sarcocystis*, *Balantidium*, *Echinostoma*, *Dicrocoelium* and *Wolbachia*. Group C differed from group B in having significantly higher signatures of mainly viral families of Poxviridae, Papillomaviridae, Coronaviridae, Bunyaviridae, Retroviridae, Herpesviridae, Reoviridae, Anelloviridae and Togaviridae and bacterial signatures of *Rickettsia* and *Legionella* compared to Group B. Group C differed from Group A in having significantly higher detection of the viral signatures of Poxviridae, Togaviridae, Papillomaviridae, Coronaviridae, Bunyaviridae, Herpesviridae, Anelloviridae, Retroviridae, Reoviridae, Parvoviridae, Rhabdoviridae, Paramyxoviridae, Arenaviridae, Picornaviridae, Circoviridae, Flaviviridae, Adenoviridae, Birnaviridae, Caliciviridae, Polyomaviridae, Orthomyxoviridae, Iridoviridae, Bornaviridae, Astroviridae; bacterial signatures of *Legionella*, *Porphyromonas*, *Lactococcus*, *Prevotella*, *Bartonella*, *Pseudomonas*, *Arcobacter*, *Helicobacter*, *Bordetella* and *Proteus*; fungal signature of *Nosema*, *Ajellomyces*, *Rhizopus*, *Cunninghamella*, *Candida*, and *Trichosporon*; and parasitic signature of *Schistosoma*, *Echinococcus* and *Hymenolepis*. The cancer samples which could not be grouped into a cluster (Singletons) showed significant differences in the detection of certain viral and microbial signatures than the rest of the clustered samples (Supplementary Table 4). The bacterial signature of *Abiotrophia* was detected significantly higher in the grouped ovarian cancer samples than the ungrouped singletons. However, in the singletons compared to the grouped samples (Group A+B+C) there was significantly higher detection of most viral signatures (except for Hepadnaviridae and Nodaviridae), bacterial signatures of *Pseudomonas*, *Lactobacillus*, *Streptococcus*, *Abiotrophia*, *Mycoplasma*, *Rickettsia*, *Bordetella* and Bacillus; fungal signatures of *Paracoccidioides*, *Ajellomyces*, *Malassezia* and *Penicillium*; and parasitic signatures of *Schistosoma*, *Entamoeba* and *Naegleria*.

### Pathochip screen validation and detection of viral insertions in human chromosomes of ovarian cancer cells

**Figure 6:**
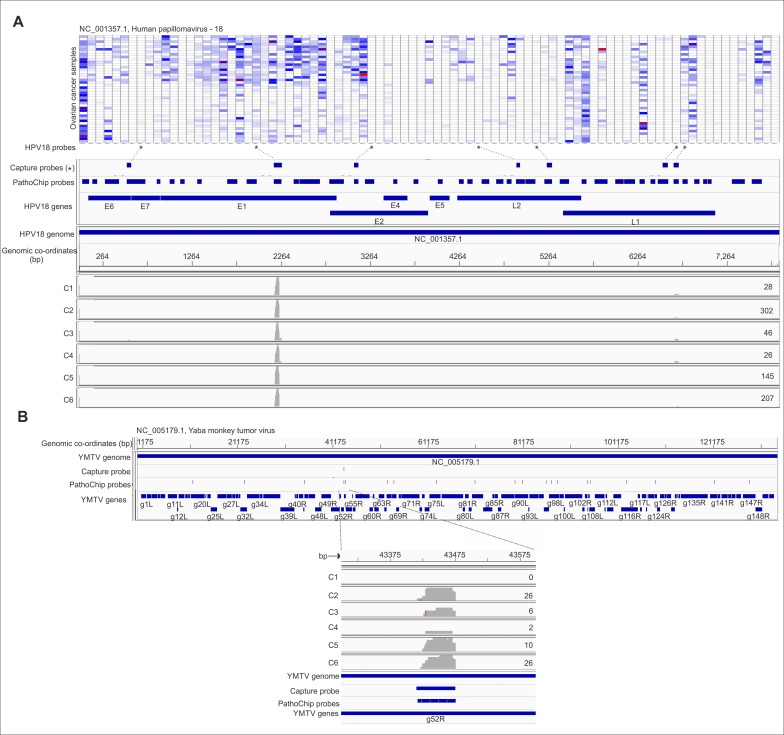
Targeted MiSeq reads align to capture probe locations Probe capture sequencing alignment is shown for individual capture pools (Capture 1-6 or, C1-6). The whole genome amplified DNA plus cDNA of the ovarian cancer samples were hybridized to a set of biotinylated probes, then captured by streptavidin beads, and used for tagmentation, library preparation and deep sequencing with paired –end 250-nt reads. The total number of MiSeq reads per capture pool for HPV18 (**A**) and Yaba Monkey Tumor Virus (**B**) are mentioned at the right end of the read coverage track. For example we obtained 302 reads for C2 capture. The miseq reads from individual capture when aligned with the metagenome of PathoChip (Chip probes) was found to cluster mostly at the capture probe regions. The genomic location are mentioned in the figure for each organism. Figure A shows the MiSeq read alignement to the HPV18 probes on the PathoChip. The probes corresponding to the HPV18 genes are mentioned. It also shows the heat map of hybridization signals of all the HPV18 probes in the PathoChip with the ovarian samples. The HPV18 probes marked (*) are the probes that were biotinylated and used for capture of the HPV18 sequences from the whole genome amplified DNA plus cDNA of the ovarian cancer samples. Figure B shows the MiSeq read alignement to the PathoChip probes for Yaba Monkey Tumor Virus. MiSeq reads aligned to the 1 capture probe used which corresponded to g52R gene of the virus.

Probes of certain viruses, which were detected positive in the PathoChip screen were used as a target reagent (Supplementary Table 5) to capture the genomic sequences of amplified products of the pooled ovarian samples. The selected targets were then subjected to next generation sequencing. The sequences, when aligned to the PathoChip metagenome, showed that they aligned at or near the capture probe locations, thus validating the PathoChip screen results (Figure 6 and Supplementary Figure 2). The sequence alignments to the PathoChip metagenome were visualized using the Integrative Genomics Viewer (IGV) program. Capture probes of Yaba Monkey Tumor virus, HTLV-2, HHV6a, Human adenovirus D, HPV16, HPV18, HPV2 and Iridovirus (Frog virus 3) also hybridized to and captured the viral sequences from the ovarian cancer samples (Supplementary Figure 2). The YMTV sequence identified the g52R ORF.

**Figure 7:**
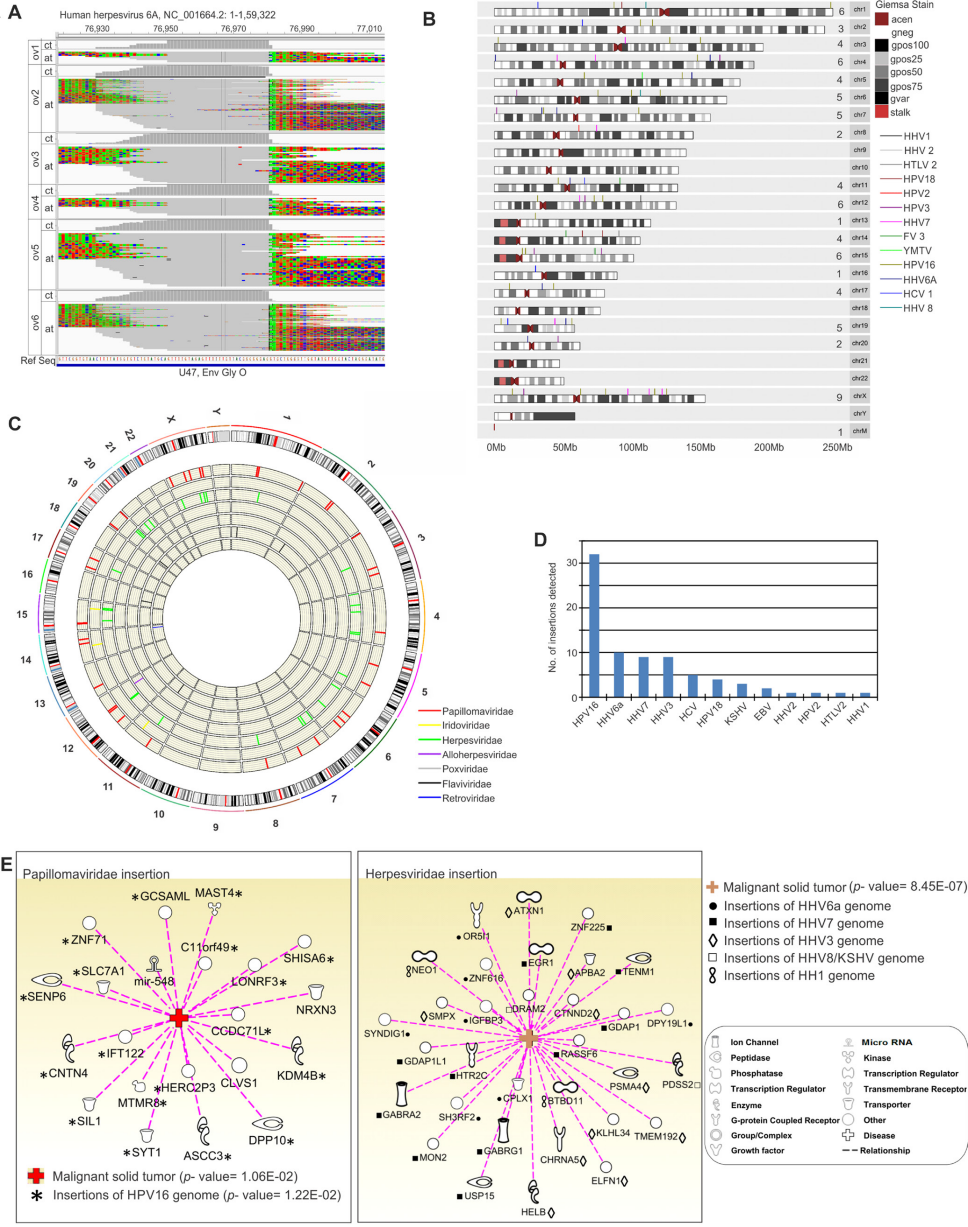
Viral genomic integrations in the host chromosome (**A**). Alignment of the MiSeq reads to the reference of HHV6A, showed soft-clipped regions that do not align to the corresponding viral reference sequences. These soft-clipped reads shown were then extracted from the alignment and mapped (containing sequences of potential pathogen-integrated human loci) to the human genome, which reveals the exact human and pathogen integration breakpoints. (**B**). Karyogram plot of virus insertion sites in human chromosomes. All the insertion sites were included. The number of insertion sites in each chromosome is mentioned in the figure before chromosome number. G-banding annotation for each chromosome is shown; gneg - Giemsa negative bands; The Giemsa positive bands have further been subdivided into gpos25, gpos50, gpos75, and gpos100 with the higher number indicating a darker stain; acen - centromeric regions; gvar - variable length heterochromatic regions; stalk - tightly constricted regions on the short arms of the acrocentric chromosomes (**C**). Circos plot highlighting fusion events for the viral insertions into individual human chromosomes. All the reads were taken into account and chromosome numbers are mentioned. Viral insertions for individual families are represented in the inner concentric circular tracks. The outermost track shows all the insertions taken together highlighting the karyotype of each chromosome. (**D**). The number of individual viral genomic insertions in human somatic chromosomes detected in the study are shown. (**E**) Association of host genes affected by viral genomic integrations to malignant tumor formation, analysed by Ingenuity Pathway Analysis (IPA) program that showed highly significant *p*- value for such association.

We also determined from our analyses that there were certain viral genomic integrations in the host chromosomes, by the Virus-Clip method described in the material and methods section (Figure 7 and Supplementary Table 6). We identified regions of some of the sequences that aligned to the PathoChip metagenome to contain soft-clipped segments, which could not be aligned to the metagenome (Figure 7A). However, these sequence segments did map to the human genome indicating specific sites of microbial genomic integrations in the human genome. We detected the highest number of viral integration sites in human chromosomes for HPV16 with over 30 integrations (Figure 7B–7D) with 5 integrations in the X-chromosome and 3 in chromosome 6 (Figure 7B, Supplementary Table 6). This was followed by HHV6a, HHV7 and HHV3 with less than 10 integrations (Figure 7B–7D, Supplementary Table 6). The genes at or proximal to which we detected the viral integrations were then subjected to Ingenuity Pathway Analysis (IPA) software [11], to determine if those genes were associated with the development of cancer (Figure 7E). The software calculates the significance of such associations.

### Identification of HPV insertions in ovarian cancer

Examination of the HPV insertion data shows integration of HPV16 genomic sequences around the polyA sequence of E5 (co-ordinate 4184–4213 of NC_001526.2), which was known to be hotspot for integration [12], integrated at intronic and intergenic regions of a number of human chromosomes (Supplementary Table 6). HPV16 integration was seen at the intronic regions of MAST4 (chr5), IFT122 (chr3), CYFIP1 (chr15), EEPD1 (chr7), C11orf49 (chr11), SYT1 (chr12), HERC2P3 (chr15), ZNF71 (chr19), ASCC3 (chr6), GCSAML (chr1), MTMR8 (chrX), SIL1 (chr5), CNTN4 (chr3), KDM4B (chr19), METTL20 (chr12), DPP10 (chr2) and SENP6 (chr6). We also detected HPV16 genomic integrations at about 29 Kb upstream of the SLC7A1 gene (chr13), 15 Kb upstream of the SHISA6 (chr17), 56 Kb upstream of the ncRNA gene LOC101928137 (chr12), 21Kb upstream of GS1-600G8.3 (chrX), 33 Kb upstream of CCDC71L (chr7), 12 Kb upstream of LONRF3 and 81 Kb downstream of ncRNA LINC01285 (chrx), 26 Kb downstream of LOC644172, and 53 Kb upstream of LRRC37A4P (chr17).

Regions from the coding sequence of the E1 gene of HPV18 were found to be integrated at the intronic regions of ncRNAs LOC100131564 (chr1) and MIR548AZ (chr14), as well as at intergenic regions of the mitochondria chromosome. Genomic regions of the L1 gene of HPV18 were also detected at the intronic region of the NRXN3 gene (chr14). Among other HPV insertions, we detected the coding sequence of the L1 gene of HPV2 at the intronic region of the CLVS1 gene in chr8. Of the 36 genes that could be affected due to HPV genomic insertions, 21 were found be significantly associated with malignant solid tumors (*p* value =1.06E-02) as predicted by Ingenuity Pathway Analysis software [11] (Figure 7E). Of the probable 32 genes that could be affected by HPV 16 genomic insertion at or near those genes, 18 of them, namely ASCC3, C11orf49, CCDC71L, CNTN4, DPP10, GCSAML, HERC2P3, IFT122, KDM4B, LONRF3, MAST4, MTMR8, SENP6, SHISA6, SIL1, SLC7A1, SYT1 and ZNF71 were found to be significantly associated with malignant solid tumors (*p*- value = 1.22E–02) (Figure 7E). Among the other HPV genomic insertions detected that could affect gene expression of 4 others, 2 genes, MIR548AZ and NRXN3 were affected by HPV18 genomic integration at the intronic region and the CLVS1 gene which was affected by intronic integration of HPV 2 were also found to be significantly associated with malignant solid tumor formation (Figure 7E).

### Herpes virus insertions within the ovarian cancer chromosomes

Among the herpesviridae genomic insertions we detected were that of HHV6a, KSHV, Herpesvirus 4, Herpesvirus 1, Herpesvirus 2, HHV3 and HHV7 (Figure 7B–7D, Supplementary Table 6). Of the 36 genes, at or proximal, we detected many herpesviral genomic integrations. 32 were significantly associated with tumorigenesis (*p*-value = 8.45E-07) as predicted by IPA software (Figure 7E). Coding sequence (CDS) of the U47 gene of HHV6a (NC_001664 at 76981) which encodes for the envelope glycoprotein O, involved in virion morphogenesis was found to be integrated at various regions of the host chromosome (chr), namely at the intronic region of SH3RF2 gene (chr 5), ZNF616 gene (chr19), SYNDIG1 gene (chr20), CPLX1 (chr4), at the exonic region of OR5I1 (chr11), at the downstream of DPY19L1 (chr7), and at certain intergenic regions like 58Kb upstream of LHX1 and 25Kb upstream of IGFBP3 (chr7) (Supplementary Table 6). Most of these genes which may be affected due to HHV6a genomic insertions at or near the genes except for LHX1 were found to be significantly associated with different cancers (*p*-value = 8.54E–04) (Figure 7E).

Many of the capture probes used were from the conserved sequences of Herpesviruses (Supplementary Table 5), and these conserved probes allowed for detection of Herpesvirus 4, Herpesvirus 1, Herpesvirus 2 genomic sequences integrated at various somatic chromosomal locations (Supplementary Table 6); CDS of ORF71 of Herpesvirus 4 was detected integrated within the intergenic region of chromosome M, genomic sequence matching to the CDS of ORF18 of Herpesvirus 1 was found integrated at the intronic region of BTBD11 (chr12), and genomic sequence of the CDS of UL42 gene which encodes the DNA polymerase processivity subunit for DNA replication was found to be integrated at the intronic region of the NEO1 gene (chr15). Both of these genes are found to be associated with endometrioid carcinoma (*p*-value = 2.27E–02) (Figure 7E).

CDS of vIRF-2 (viral interferon regulatory factor 2) of HHV8 was found to be integrated 57Kb downstream of DRAM2 (chr 1), while tegument protein coding sequence was seen to be integrated at the intronic region of the PDSS2 tumor suppressor gene (chr6) [13] (Supplementary Table 6). Again, both of these genes were associated with cancer (Figure 7E).

Interestingly, we detected CDS of ORF6 that encodes the helicase-primase subunit for DNA replication of the HHV3 sequence integrated at multiple sites of different chromosomes (Supplementary Table 6). This region could be a hotspot for HHV3 integrations within the host chromosomes. We detected insertions at the intronic regions of TMEM192 (chr4), ATXN1 (chr6), APBA2 (chr15), CTNND2 (chr5), upstream of HELB (chr12), at a position that is just upstream of CHRNA5 and downstream of PSMA4 (chr15), as well as at certain intergenic regions in certain chromosomes. We detected intergenic insertions which includes regions 13 Kb downstream of SMPX and 34Kb upstream of KLHL34 in X chromosome, 10Kb upstream of ELFN1 and 82Kb downstream of TFAMP1 (chr7). Except for TFAMP1, all other genes are found to be associated with epithelial cancer (*p*- value = 2.11E–03) (Figure 7E).

Similar to the HHV3 data, we detected a specific region of the HHV7 genome to be integrated at multiple sites in the chromosomes (Figure 7B–7C, Supplementary Table 6). The CDS of the U30 gene of HHV7, encoding the tegument protein UL37 that helps in virion morphogenesis was found to be integrated at the intronic or intergenic region of certain chromosomes. We detected HHV3 insertions at the intronic regions of ZNF225 (chr19), TENM1 (chrX) and HTR2C (chrX), and also at certain intergenic regions, some of which are less than 35Kb from the affected genes. Therefore, this may have an effect on promoting or suppressing the transcription of those genes. For example, we detected insertions 17 Kb downstream of RASSF6 and 26 Kb downstream of LOC728040 in chromosome 4; 32 Kb downstream of GDAP1 (chr8); 11 Kb downstream of USP15 and 46Kb upstream of MON2 (chr12); 35Kb downstream of GABRA2 and 90 Kb upstream of GABRG1 (chr4). Except for LOC728040, the other genes having HHV7 genomic insertions at or in their proximity were seen to be significantly associated with adenocarcinoma (*p*- value = 2.33E–04) (Figure 7E).

### Insertions detected for retrovirus, hepadnavirus, yaba monkey tumor virus and frog virus 3

Among the other viral insertions detected were HTLV-2 (Supplementary Table 2), whose genomic region encoding gag-pro-pol was detected at the intronic region of CCDC88C (chr14). The 3′UTR region of HCV was detected at the intronic, intergenic as well as downstream of certain genes in a number of chromosomes. We detected insertion at the intronic region of RBM4 (chr11), known to be associated with cancer [14] and ncRNA SMG1P5 (chr16), downstream of TINAGL1 (chr1) and LOC339807 (chr2) and at an intergenic region that is 30Kb upstream of ZNF846 and 11Kb downstream of FBXL12 in chromosome 19. Interestingly, we also detected Yaba Monkey Tumor Virus (YMTV) genomic sequences encoding the G protein-coupled chemokine receptor-like protein at the intergenic region of a number of genes in chromosome 5 (Supplementary Table 2). We also detected Alloherpesviridae genomic sequence (Frog virus 3) insertions in host chromosomes. CDS of FV3gorf8R gene encoding the largest sub-unit of DNA-dependent RNA polymerase II of Frog virus 3 was inserted at the intronic region of FAT3 gene (chr11), upstream of PTGDR gene (chr14), 86Kb downstream of C15orf59-AS1 and 18Kb upstream of TBC1D21 gene (chr15). FAT3 gene and PTGDR gene, both are shown to be associated significantly (*p*- value = 8.41E–04) with esophageal adenocarcinoma by IPA analysis.

## DISCUSSIONS

We previously reported 2 distinct microbial signatures specifically associated with triple negative breast cancer [6]. In the present study we used the same pan-pathogen array technology to detect the microbial signatures associated with ovarian cancers [6, 9]. Evidences from a number of studies have indicated that the mutualistic or pathogenic resident or transient viruses, bacteria, fungi and parasites in our body may increase our potential cancer risk. In this regard it has been shown that differences in the microbiome in an individual can correlate with different susceptibility to diseases [7, 15, 16]. Apart from inducing cancer, the microbiome may also influence the course of the cancer. However, it is also possible that the tumor microenvironment provided a specialized niche in which a specific microbiome can persist. In either case establishing the unique microbiome of different cancers may provide biomarkers, as well as insights for diagnosis, prognosis, prevention and the development of treatments for microbe-associated cancers.

We selected those signatures in cancer samples with adjusted *p* value < 0.05 (adjusted by the Benjamini–Hochberg procedure), logFC > 0.5. Under the adjusted *p*-value cutoff, we did not observe much significant ones present in either of the controls. To provide information as to what are present in control, we present the top ones and used nominal *p*-value < 0.05 as cutoff for the controls without any multiple comparison correction. Including these nominally significant signatures present in controls would provide us some suggestive evidence of detection, though caution should be used for potential false positives brought by multiple comparison.

Our data show that the microbiome of ovarian tumors is quite different from its surrounding non-cancerous tissue and very different from ovarian tissue that has never been in the proximity of a tumor. A defining ovarian tumor microbiome signature does emerge from the data. The microbiome we detected is robust and, for some organisms, unexpected. However, we are using a very sensitive approach [9] which can detect not only low levels of specific viruses and microorganisms but also related members of a viral or microbial family that have yet to be characterized. Thus in cases where, for example, an unexpected organism is detected it may be a related family member that has yet to be characterized in human flora.

We detected a large number of bacterial signatures that were significantly detected in cancer versus the non-matched controls, as mentioned above, the tumor micro-environment may create a milieu favorable to the persistence for many bacteria. Only a few studies have suggested an association of bacteria with ovarian cancer. One case report showed an association of *Brucella* [17]; another has found that 70% of the ovarian cancer tissues contained *Chlamydia* infection which was not seen in healthy controls [8]. *Chlamydia* is known to contribute to cancer by inhibiting apoptosis, inducing DNA damage response and increasing susceptibility to other infections [8]. *Mycoplasma* has also been found associated with 59% of the ovarian cancer tissues tested [18]. We detected *Brucella*, *Chlamydia* and *Mycoplasma* in 76%, 60% and 74%, respectively, of the ovarian cancer samples screened.

Among the bacterial genera detected in the ovarian cancer, many have been reported earlier to be either associated with other types of cancers [19–31], for example: *Streptococcus*, *Staphylococcus*, *Bacillus*, *Mycoplasma*, *Chlamydophila* in lung cancer [19, 22]; *Pediococcus* in pancreatic cancer [19, 23]; *Staphylococcus, Mycoplasma and Chyseobacterium* in breast cancer [19, 24, 25]; *Fusobacterium* and *Prevotella* in oral cancer [19, 26] ; *Salmonella* in gall bladder cancer [19, 27]; *Chlamydia* in Pulmonary Mucosa-Associated lymphoid tissue lymphoma [19, 28]; *Streptococcus*, *Fusobacterium*, *Escherichia* and *Mycoplasma* in colorectal cancer [19, 25, 29–31].; as well as *Treponema* and *Streptococcus* in oesophageal cancer [21].

Fungal infections in immunocompromised cancer patients are important causes for morbidity and mortality, and are a major therapeutic challenge. Thus an association of yeast and zygomycetous fungal infections with cancer may be expected. There have been reports of infection with *Aspergillus, Candida, Rhizomucor*, *Cladosporium*, *Acremonium*, *Alternaria*, *Cryptococcus*, *Pneumocystis*, *Coccidiodes*, *Trichosporon*, *Malassezia*, *Rhodotorula* and *Geotrichum* in different cancer patients [32–36]: all of these have been detected in the ovarian cancers in our study, with the highest signal intensity detected with the probes for *Cladosporium* in all the cancer samples.

Certain parasitic worms can also raise the risk of cancer. For example, infection with *Schistosoma* is associated with several cancers [37–39]. In their hosts, parasites establish long-term chronic infections and significantly downregulate the host immune response [40]. We detected molecular signatures of a number of parasites in the ovarian samples, some of them quite surprising, but may suggest that sub-clinical infections may be more prevalent than presently known. The molecular signatures for the zoonotic parasite *Dipylidium* were detected with the highest hybridization signal in all the ovarian cancer samples screened. Although there have been reports of *Dipylidium* infection in humans [41, 42], there have been no reports of an association with cancers. *Trichuris* was detected with high hybridization signal in all the ovarian cancers screened. This correlates with our earlier study where *Trichuris* was detected in 96% of triple negative breast cancer samples [6]. There have been other reports which demonstrated an association of *Trichuris* with cancer [43, 44]. Epithelial dysregulation and hyper proliferation during chronic infection of *Trichuris* [45] has also been reported, which potentially could promote tumorigenesis. The association of other parasites like *Echinococcus, Strongyloides*, *Trichinella*, *Schistosoma, Leishmania*, *Ascaris*, *Trichomonas* to cancer was not unique to our study, and has been previously reported [44, 46–50].

Our study shows a significant association of molecular signatures of 10 viral families with ovarian cancer. Among these were specific signatures for parapox and pox viruses including Yaba Monkey tumor virus, Yaba-like disease virus, Monkeypox virus and Myxoma virus. There have been no reports of parapox and pox virus association with ovarian cancer; however, the signatures of various monkey pox viruses suggest that we may be detecting a heretofore uncharacterized human variant.

One of the most intriguing aspects of our viral data is the finding of widespread integration of viral sequences into the genome of the tumor tissue. Several studies have demonstrated an association of HPV with ovarian cancers [2, 3, 51]. We detected molecular signatures of high risk HPV16 and 18 along with other low risk HPVs in the ovarian cancer samples screened. Interestingly, molecular signatures of only low risk HPVs were found associated with the non-cancerous controls potentially implicating the high risk viruses with the origin or propagation of the cancer. In this regard, integration of HPV genomic regions into the human genome has been considered an important event in cancer development. We have detected the HPV16 genomic integration hotspot, which is located around the polyA sequence of the HPV16 gene for E5 [12], to be integrated at various intronic regions, as well at intergenic regions that are within 56Kb upstream of a number of cancer related human genes. It is known that insertions, or genomic perturbations, within 100Kb upstream of a gene can affect gene expression [52, 53], thus the insertions we detect could deregulate gene expression. In this regard, Ingenuity Pathway Analysis (IPA) showed that many of the genes potentially affected by HPV insertion can be associated with cancer.

Integration of HPV also often occurs within the E1 or E2 regions. These regions become transcriptionally inactivate after integration due to disruption of the open reading frames [54], this is believed to be a prerequisite for oncogenesis. In our study, we have detected the coding sequences of the E1 gene of HPV18 to be integrated at the intronic regions of non-coding RNA genes in the host chromosomes. This ncRNA disruption may potentially play a role in the development or progression of ovarian cancer [55]. Other than HPV16 and 18 genomic integrations, we also detected integration of other low risk HPVs, which were again integrated at or near genes significantly associated with cancer.

Research on viral associations with ovarian cancer, other than HPVs, is very limited. Several studies have shown an association of Polyomaviruses [56] with ovarian cancer. Additionally herpesviruses [8, 57], and Retroviruses (Mouse mammary tumor virus-like DNA) [58, 59] have been detected in 50% and 16%, respectively, of ovarian cancers. In the present study, we not only detected specific molecular signatures of herpesviruses HHV4, HHV8, HHV5, HHV6a and HHV6b in ovarian cancer samples, but also detected the HHV6a hypervariable U47 region integrated at exonic, intronic and intergenic regions, as well as upstream regions of certain human genes, and at sub-telomeric regions of chromosomes 4 (Supplemental Table 6). There have been reports of HHV-6A and HHV-6B viral genome integration, mostly in the telomeric/sub-telomeric region of several host chromosomes [60, 61]. Since several diseases, including cancer, are associated with telomere dysfunction, genomic integration of HHV-6a at the telomeric region could be a contributing factor to ovarian cancer.

HHV6a integration was also detected at a number of significant genomic sites that may relate to the genesis of ovarian cancer: 1) HHV6a sequences were found integrated 25Kb upstream of the IGFBP3 gene that encodes an IGF-binding protein 3 (IGFBP). Insulin-like growth factors (IGFs) are mitogens that play an important role in regulating cell proliferation and anti-apoptosis and thus promotes cancer [62]. Recent studies suggest that increased levels of IGF-I are associated with increased risk for several common cancers including breast [63], prostate [64], lung [65], and colorectum [66] cancers. IGF-binding proteins (IGFBPs) can influence the actions of IGFs [62]. IGF-binding protein 3 (IGFBP3), is a major IGF-I-binding protein that suppresses the mitogenic action of IGF-I [62]. Thus genomic perturbation upstream of IGFBP3 by HHV6A could lead to increased mitogenic action of IGF-1. 2) HHV6a sequences were found integrated in the intronic region of SH3RF2, an oncogene that is over-expressed in human cancers and regulates p21-activated kinase 4 (PAK4) protein stability. The viral genomic integration at the intronic region of the gene may enhance the oncogene expression [67]. The integrations which results in human-viral fusion transcript may also lead to increased expression of a gene, as has been reported for HBV integration in hepatocarcinoma [68].

Other herpesvirus integrations sites of interest include the insertion of the coding sequences of the HHV1 UL42 gene within the intronic region of the NEO1 gene which expresses neogenin. Low expression of neogenin has been found in a variety of human cancers, such as pancreatic [69], colon cancer [70], esophageal squamous cell carcinoma [71], gliomas [72] and breast cancer [73]. Altered expression of neogenin leading to loss of pro-apoptotic activity can contribute tumorigenesis [74]. Additionally the coding sequences of the KSHV vIRF-2 gene were found integrated 57Kb downstream of the Damage-regulated autophagy regulator 2 (DRAM2). This may affect its role as an effector molecule for p53-mediated apoptosis. It is already known that DRAM2 is down-regulated in ovarian tumors and reduced expression of DRAM2 may contribute to anti-apoptosis in tumor cells [75]. The coding sequences of HHV3 ORF6, the helicase-primase subunit for DNA replication [76], was detected at 9 separate sites in different chromosomes (Supplementary Table 6), thus it could be an HHV3 hotspot for integration. Similarly, we detected coding sequences of the HHV7 U30 gene, the UL37 tegument protein [77], integrated at multiple sites in different chromosomes, which again could be an integration hotspot for HHV7. The supplemental data contains all of the integration site data for all viruses tested.

In conclusion, our data suggests that due to the nature of ovarian tumor and its micro-environment, significant perturbations have occurred in the ovarian microbiome, resulting in a specific ovarian tumor microbiome signature. These changes may relate to the genesis or propagation of the cancer, alternatively the tumor micro-environment may provide a favorable milieu for these micro-organisms to persist. We feel that these data provide a valuable biomarker for ovarian cancer which, when correlated with patient treatment and outcome data, may be diagnostic, prognostic and guide treatment approaches. Further, understanding the contributions of these signatures may guide additional research activities into the molecular pathogenesis of ovarian cancer.

## MATERIALS AND METHODS

### Study samples

The study was approved by the institutional review board at the University of Pennsylvania (Protocol number 819358). The computerized records at the a) Tumor Tissue and Biospecimen Bank and b) the clinical archives of the Department of Pathology and Laboratory Medicine were searched and a total of 99 primary and recurrent or metastatic tumors of ovarian origin were identified (Supplementary Table 1). Both the metastatic or recurrent tumor were still of ovarian origin. Histology of the cases evaluated included malignant surface epithelial tumors (serous, endometrioid, mucinous, clear cell, transitional cell, mixed types and carcinosarcoma) and 1 case of small cell carcinoma, hypercalcemic type. The matched control tissues were non-tumor ovarian tissue from ipsilateral or contralateral ovary from 20 ovarian cancer patients (Supplementary Table 1). The non-matched control benign tissues were from prophylactic oophorectomy surgery in women with BRCA mutations.

The original H&E slides were reviewed and one representative formalin-fixed, paraffin-embedded tissue block was chosen per case and cut. Tumors needing macro-dissection were received in the form of 10μm sections on glass slides with marked guiding H&E slides, while tumors that did not require macro-dissection were received as 10 μm paraffin rolls.

### PathoChip design, sample preparation and microarray processing

The PathoChip Array design has been previously described in detail [6, 9]. Briefly, the probes were generated in silico from a metagenome of 58 chromosomes comprising the genomes of all known viruses as well as known human bacterial, parasitic and fungal pathogens [9]. PathoChip comprises 60,000 probe sets manufactured as SurePrint glass slide microarrays (Agilent Technologies Inc.), containing 8 replicate arrays per slide. Each probe is a 60-nt DNA oligomer that targets multiple genomic regions of the viruses and higher pathogens [9].

PathoChip screening was done using both DNA and RNA extracted from formalin-fixed paraffin-embedded (FFPE) tumor tissues as described previously [6, 9]. 99 de-identified FFPE samples of invasive epithelial malignant tumors of ovarian origin were received as 10 μm sections on non-charged glass slides from the Abramson Cancer Center Tumor Tissue and Biosample Core. Additionally, 20 matched and 20 non-matched control samples were provided as paraffin rolls. Matched controls were obtained from the adjacent non-cancerous ovarian tissue of the same patient from which the cancer tissues are obtained, non-matched controls were the ovarian tissues obtained from non-cancerous individuals. DNA and RNA were extracted in parallel from 5 rolls or mounted sections of each FFPE sample. The quality of the extracted nucleic acids was determined by agarose gel electrophoresis and the A260/280 ratio. The extracted RNA and DNA samples were subjected to whole transcriptome amplification (WTA) as previously described [6, 9]. RNA and DNA from 40 cancer samples were subjected to WTA individually, and the rest were pooled in groups of 4–5 samples together, so that 99 samples were screened in 54 arrays. 20 of each type of controls were also pooled in groups of 5 for the WTA step, so that we have 4 arrays for each of the control types. The WTA products were analyzed by agarose gel electrophoresis. Human reference RNA and DNA were also extracted from the human B cell line, BJAB and were used for WTA as previously described [6, 9]. The WTA products were purified, (PCR purification kit, Qiagen, Germantown, MD, USA); the WTA products from the ovarian cancers were labeled with Cy3 and those from the human reference DNA were labeled with Cy5 (SureTag labeling kit, Agilent Technologies, Santa Clara, CA). The labeled DNAs were purified and hybridized to the PathoChip as described previously [6, 9]. Post-hybridization, the slides were washed, scanned and visualized using an Agilent SureScan G4900DA array scanner [6, 9].

### Microarray data extraction and statistical analysis

The microarray data extraction and analyses have been described previously [6, 9]. The raw data from the microarray images were extracted using Agilent Feature Extraction software [6, 9]; Apart from the previously described method we also used the R program for normalization and data analyses [78]. We calculated scale factor using the signals of green and red channels for human probes. Scale factors are the sum of green/sum of red signal ratios of human probes. Then we used scale factors to obtain normalized signals for all other probes. For all probes except human probes, normalized signal is log2 transformed of green signals / scale factors modified red signals (log2 g – log2 scale factor * r). On the normalized signals, *t*-test is applied to select probes significantly present in cancer samples by comparing cancer samples versus controls (un-matched and matched controls) and to select probes significantly present in un-matched or matched controls versus the cancer samples. The significance cutoff was log2 fold change > 0.5 and the adjusted *p*-value < 0.05. The adjusted *p*-values were obtained for multiple corrections by using the Benjamini–Hochberg procedure [79]. We detected no significant ones in control under this adjusted *p*-value cutoff. So we present the top ones in control with nominal *p*-value < 0.05 without any multiple comparison correction, in order to have a comparison with the significant ones present in cancer samples. Prevalence was calculated based on the detection of the signatures in the cancer and the control samples as percentage.

The cancer samples were also subjected to hierarchical clustering, based on the detection of microbial signatures in the samples, using the R program (Euclidean distance, complete linkage, non-adjusted values) [78, 80], and the clusters were validated by CH index (Calinski and Harabasz index) which is implemented in R package as NbClust [81]. CH index is a cluster index that maximize inter-cluster distances and minimize intra-cluster distances. We calculated the possible cluster solution that would maximize the index values to achieve the best clustering of the data. The significant differences between the clusters observed by these methods were determined using *t*-test. Additional topological-based data analyses were conducted using the Ayasdi software (Ayasdi, Inc.), (using Euclidean (L2) metric, and L-infinity centrality lens), where statistical significance between different groups was determined using the two-sided *t*-test.

### Probe capture and next generation sequencing

Probe Capture method has been previously described [6, 9]. Briefly, selected PathoChip probes that identified microbial signatures in the ovarian cancer samples were made as biotinylated derivatives and used to capture the microbial target nucleic acid from pooled WTA products from the ovarian cancer samples. Hybridization was followed by capturing the targeted sequences using Streptavidin coated magnetic beads as previously described [6, 9]. The libraries of the targets were generated for NGS using Nextera XT sample preparation kit (Illumina, San Diego, CA, USA) [6, 9]. 6 libraries were generated, ov1-6. The selected probes used for the target capture are listed in (Supplementary Table 5). The libraries were submitted to the Washington University Genome Technology Access Center (St. Louis, MO) for quality control measurements, library pooling, and sequencing using an Illumina MiSeq instrument with paired-end 250-nt reads. Adapters and low-quality fragments of raw reads were first removed using the Trim Galore software (http://www.bioinformatics.babraham.ac.uk/projects/trim_galore/). The processed reads were then aligned to the PathoChip metagenome and the human genome using Genomic Short-read Nucleotide Alignment Program (GSNAP) [82] with default parameters. Post alignment feature Counts [83] was employed to count the number of reads aligned to each of the capture probe regions, and visualized in IGV [84] (Figure 6).

### Virus fusion identification

Prior to fusion detection, raw reads were trimmed in order to remove adapters and low-quality fragments by Trim Galore software (http://www.bioinformatics.babraham.ac.uk/projects/trim_galore/). We then used Virus-Clip [85] to identify the virus fusion sites in the human genome. Specifically, the virus genome was used as the primary read alignment target, and first aligned the reads to the PathoChip metagenome. Some of the mapped reads contained soft-clipped segments, which were then extracted from the alignment (potentially containing sequences of pathogen-integrated human loci) and mapped to the human genome. Using this mapping information, we could pinpoint the exact human and pathogen integration breakpoints at single-base resolution. All the integration sites were then automatically annotated with the affected human genes and their corresponding gene co-ordinates from the human genome maps.

The affected host genes at or near the viral genomic integration sites were analyzed by Ingenuity Pathway software to determine if there were any significant association with cancer [11].

## SUPPLEMENTARY MATERIALS FIGURES AND TABLES














