# Supplementary material for: The ovarian cancer oncobiome
Source: Oncotarget. 2017 Mar 30;8(22):36225–45. doi: 10.18632/oncotarget.16717 (PMC5482651; doi:10.18632/oncotarget.16717)
Supplement: Supplementary file 1 [file oncotarget-08-36225-s001.pdf]

The ovarian cancer oncobiome

Supplementary Materials

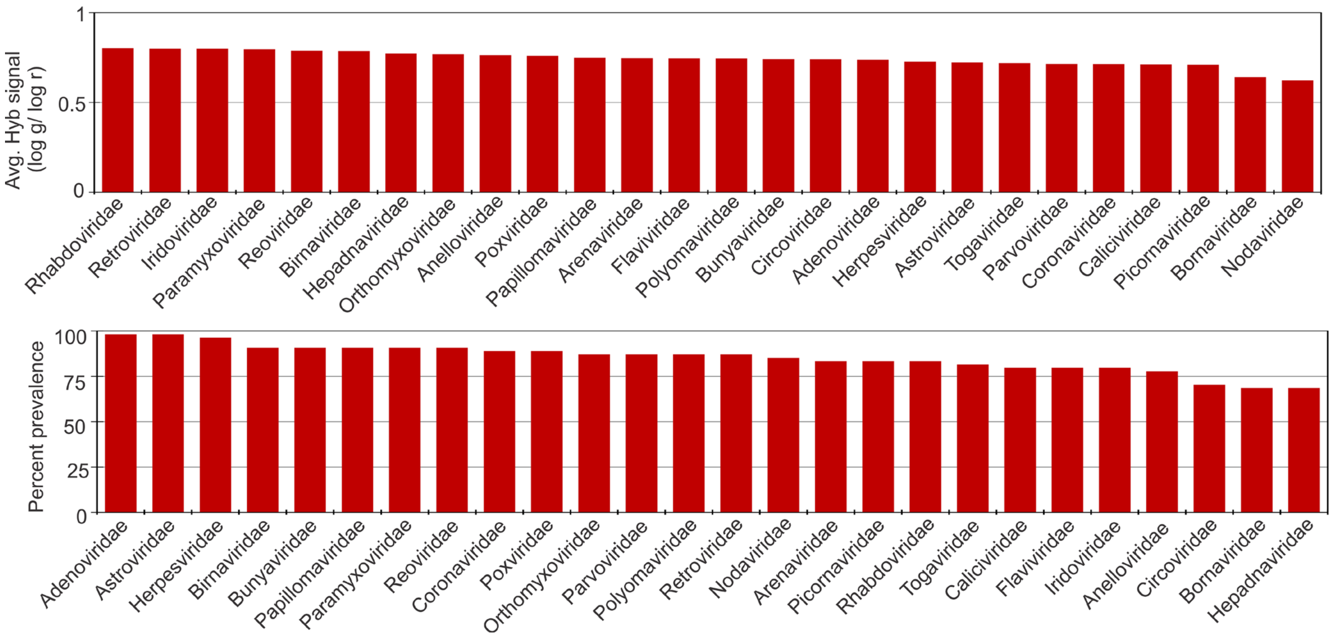

Supplementary Figure 1: Molecular signatures of viral families detected in ovarian cancer represented in bar graphs according to decreasing average hybridization signal and prevalence.

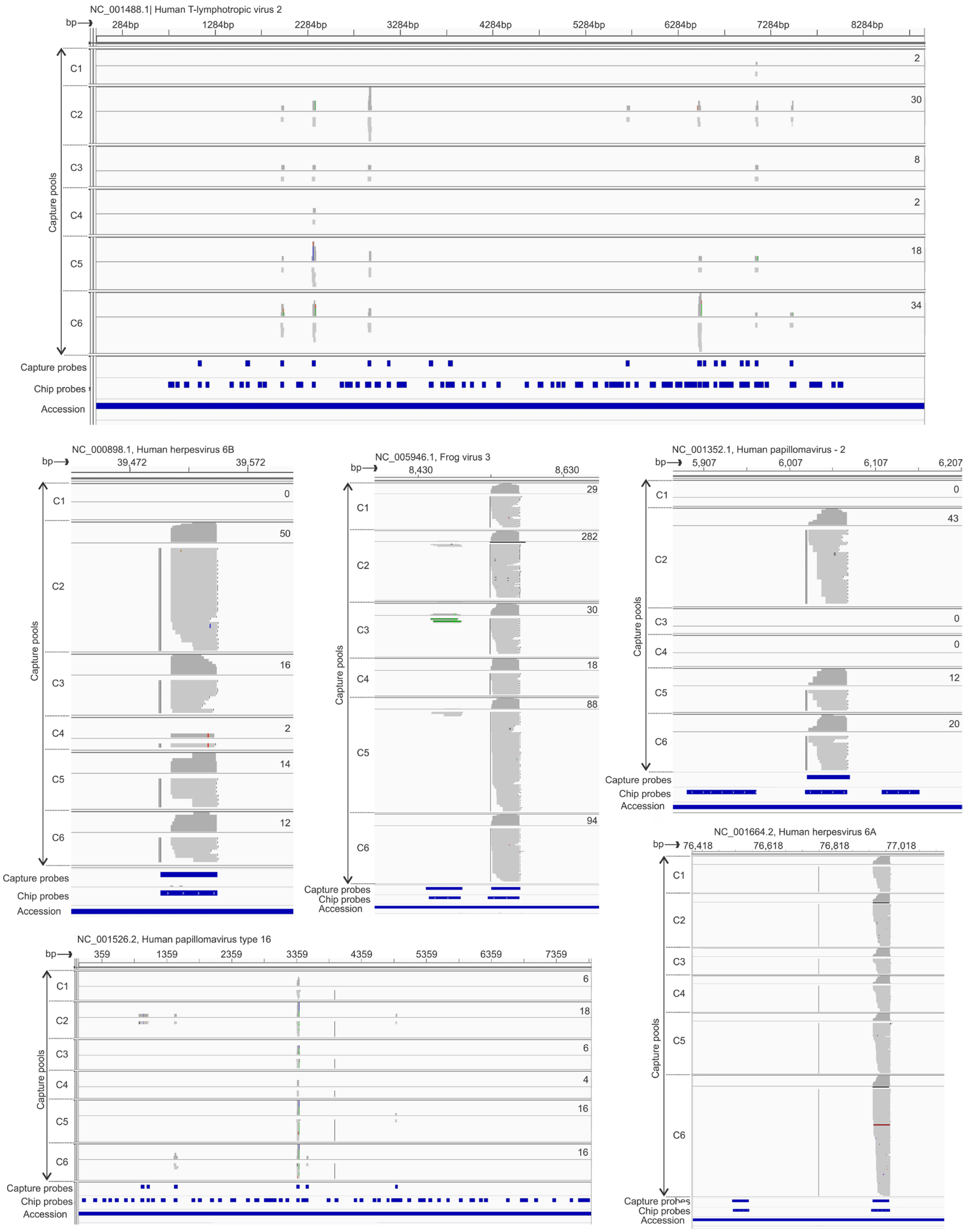

Supplementary Figure 2: Probe capture sequencing alignments post MiSeq. The MiSeq reads from individual capture (C1-6) when aligned with the metagenome of PathoChip (Chip probes) was found to cluster mostly at the capture probe regions. The genomic location along with the number of MiSeq reads are noted.

Supplementary Table 1: Available clinical details of the 99 ovarian cancer samples screened are listed. Whether the tumor specimens are from primary, metastatic or recurrence are provided, with their pathological diagnosis and age at the time of surgery. See Supplementary_Table_1

Supplementary Table 2: Specific and conserved viral probes detected significantly in ovarian cancers or controls, with their respective average hybridization signals (hyb) in ovarian cancers (OC), matched controls (MC) and non-matched controls (NC), along with their probe sequences, the origin of the sequences (name of the virus), the blast results of the probe sequences and their respective *p*-value of significance for their detection as calculated by one-sided *t*-test are mentioned. Case. *p*-value, case.adj. *p*-value are case vs non-matched control *p*-value and its respective adjust *p*-value (corrected for multiple testing); NC. *p*-value, NC.adj. *p*-value are non-matched control vs case *p*-value and its respective adjust *p*-value (corrected for multiple testing); MC. *p*-value, MC.adj. *p*-value are match control vs case control *p*-value and its respective adjust *p*-value (corrected for multiple testing). The significant values are bold faced. See Supplementary_Table_2

Supplementary Table 3. Statistical significance between ovarian cancer samples of Clusters 1, 2 and 3 obtained by NBClust software. The significant differences between the clusters observed were determined using two-sided *t*-test. See Supplementary_Table_3

Supplementary Table 4. Statistical significance between ovarian cancer samples of Groups A, B, C and singletons that are obtained by topological-based data analyses using Ayasdi software. The significant differences between the clusters observed were determined using two-sided *t*-test. See Supplementary_Table_4

Supplementary Table 5: List of the capture probe sequences used for capture sequencing. See Supplementary_Table_5

Supplementary Table 6: Gene fusion results. List of viral genomic regions integrated either in intronic, exonic, downstram or upstream of certain human genomic regions. Point of integration with genomic co-ordinates, both for the pathogen and human genome are mentioned. The numbers of reads for such kind of fusion are also mentioned. The description of the gene fusion results are as follows: Column A: type of left element of fusion fragment (micro-organism or human), Column B: left chromosome name (pathochip genome or human), Column C: left start co-ordinate, Column D: left sequence, Column E: type of right element of fusion fragment (micro-organism or human), Column F: right chromosome name (pathochip genome or human), Column G: right start co-ordinate, Column H: Host chromosomal integration site, Column I: right sequence, Column J: number of reads supporting fusion; Column K: Gene_region, indicating region (like intergenic, intronic, exon etc) of the fusion site in human genome; Column L: affected human gene name; Column M: Virus_ coordinate of fusion site in pathochip metagenome; Column N: Accession number of microorganism integrated (description of accession); Column O: Capture experiment number; Column P: Viral accession start; Column Q: Viral genomic co-ordinate at the integration start point; Column R: Region of viral genome inserted; Column S: Viral family.
See Supplementary_Table_6
